# Dataset on the RETRO-BMC cruise onboard the R/V Hespérides, April 2017, Brazil-Malvinas Confluence

**DOI:** 10.1016/j.dib.2020.105412

**Published:** 2020-03-21

**Authors:** Dorleta Orúe-Echevarría, Josep L. Pelegrí, Paola Castellanos, Carles Guallar, Humberto Marotta, Cèlia Marrasé, Jacobo Martín, Marta Masdeu-Navarro, Guillermina F. Paniagua, Jesús Peña-Izquierdo, Joan Puigdefábregas, Belén Rodríguez-Fonseca, Elena Roget, Miquel Rosell-Fieschi, Jordi Salat, Joaquín Salvador, Ignasi Vallès-Casanova, Montserrat Vidal, Álvaro Viúdez

**Affiliations:** a Departament d'Oceanografia Física i Tecnológica, Institut de Ciències del Mar, CSIC, Unidad Asociada ULPGC-CSIC, Barcelona, Spain; b Centro de Ciências do Mar e do Ambiente, Universidade de Lisboa, Lisboa, Portugal; c Department de Biologia Evolutiva, Ecologia i Ciències Ambientals, Universitat de Barcelona, Barcelona, Spain; d Ecosystems and Global Change Laboratory / International Laboratory of Global Change, Biomass and Water Management Research Center, Graduate Program in Geosciences (Environmental Geochemistry), Universidad Federal Fluminense, Niterói, Rio de Janeiro, Brazil; e Departament de Biologia Marina i Oceanografia, Institut de Ciències del Mar, CSIC, Barcelona, Spain; f CADIC-CONICET, Ushuaia, Argentina; g Centro de Investigaciones del Mar y la Atmósfera (CIMA/CONICET-UBA), and UMI-IFAECI/CNRS-CONICET-UBA, Buenos Aires, Argentina; h Departamento de Física de la Tierra, Astronomía y Astrofísica, Universidad Complutense de Madrid, Madrid, Spain; i Department de Física, Universitat de Girona, Girona, Spain

**Keywords:** Brazil-Malvinas Confluence, Hydrographic data, SeaSoar data, Microstructure data

## Abstract

This dataset, gathered during the RETRO-BMC cruise, reports multiple-scale measurements at the Confluence of the Brazil and Malvinas Currents. The cruise was carried out between 8 and 28 April 2017 onboard R/V Hespérides, departing from Ushuaia and arriving to Santos. Along its track, the vessel recorded near-surface temperature and salinity, as well as the horizontal flow from 20 m down to about 800 m. A total of 33 hydrographic stations were completed in a region off the Patagonian Shelf, within 41.2°S–35.9°S and out to 53.0°W. At each station, a multiparametric probe and velocity sensors were deployed inside the frame of a rosette used to collect water samples at selected depths; these samples were later used for several water analyses, including inorganic nutrient concentrations. Microstructure measurements were carried out in 11 of these hydrographic stations. In addition, two high-resolution three-dimensional surveys were conducted with an instrumented undulating vehicle between 40.6°S–39.0°S and 55.6°W–53.8°W. Lastly, eight high-frequency vertical profilers were deployed in the region and five position-transmitting drifters were launched. These data allow the description of the Confluence from the regional scale to the microscale, and provide a view of the variability of the frontal region on time scales from days to weeks.

Specifications table

**Table:** 

*Subject*	Environmental sciences.
*Specific subject area*	Oceanographic cruise data.
*Type of data*	Tables, figures.
*How data were acquired*	SBE 911 Plus multi-parametric probe, SBE 43 oxygen sensor, 4-beam 300 kHz RDI Workhorse Monitor, 75 kHz Teledyne RD Instruments VADCP, SE 21 SeaCAT thermosalinograph, Chelsea Group Technology SeaSoar, ARVOR-I and APEXi vertical-profiling floats, 100-m drifting buoys, Sea & Sun Technology MSS90L microstructure profiler.
*Data format*	Raw.
*Parameters for data collection*	Survey based on the position of the Brazil-Malvinas Confluence Front, derived from in-situ data, satellite images and high-resolution operational models.
*Description of data collection*	Deployment of several instruments during the RETRO-BMC oceanographic cruise between April 8 and 28, 2017, and analysis of collected water samples.
*Data source location*	Off the Patagonian continental shelf, within 41.2°S–35.9°S and out to 53.0°W.
*Data accessibility*	Repository name: Digital CSIC. Direct URL to data: http://hdl.handle.net/10261/188363
*Related research article*	D. Orúe-Echevarría, P. Castellanos, J. Sans, M. Emelianov, I. Vallès-Casanova, J.L. Pelegrí. Temperature spatiotemporal correlation scales in the Brazil-Malvinas Confluence from high-resolution in situ and remote sensing data. Geophys. Res. Lett. 46 (2019) 13,234–13,243. https://doi.org/10.1029/2019GL084246

## Value of the data

• These data present a high-resolution quasi-synoptic multi-scale oceanographic data from an intensive survey at the Brazil-Malvinas Confluence (BMC) on April 2017.
• Data include the first survey of the BMC with a SeaSoar, allowing an unprecedented description of the subsurface frontal structure and its spatiotemporal variability.
• This dataset benefits other researchers studying the physical and biogeochemical processes taking place in the BMC, from the microstructure to the regional scale.
• Data can also be used for the validation of both process-oriented and operational numerical models.

## 1 Data description

**Fig. 1:**
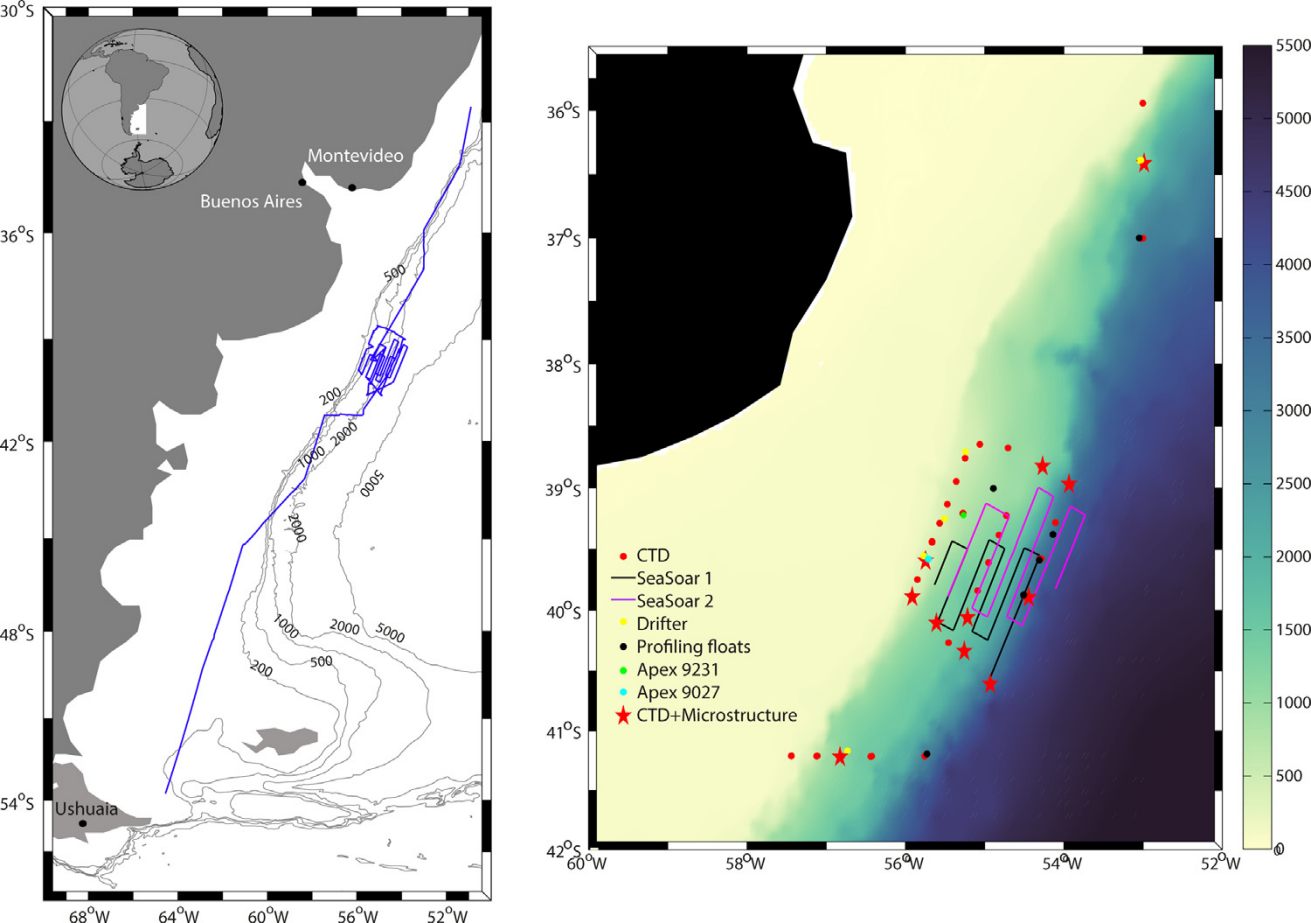
(Left) Track of the vessel during RETRO-BMC (blue line) with the 200, 500, 1000, 2000 and 5000 m isobaths (gray contours). (Right) Position of the CTD stations (red dots), SeaSoar1 and SeaSoar2 transects (black and magenta respectively) and the launching sites of the high-frequency ARVOR-I profiling floats (black dots), the APEX 9231 and APEX 9027 profiling floats (green and cyan dots), and the sub-surface drifters (yellow dots), on top the bathymetry (smoothed GEBCO, 2008, color coded in meters). (For interpretation of the references to color in this figure legend, the reader is referred to the web version of this article.)

**Fig. 2:**
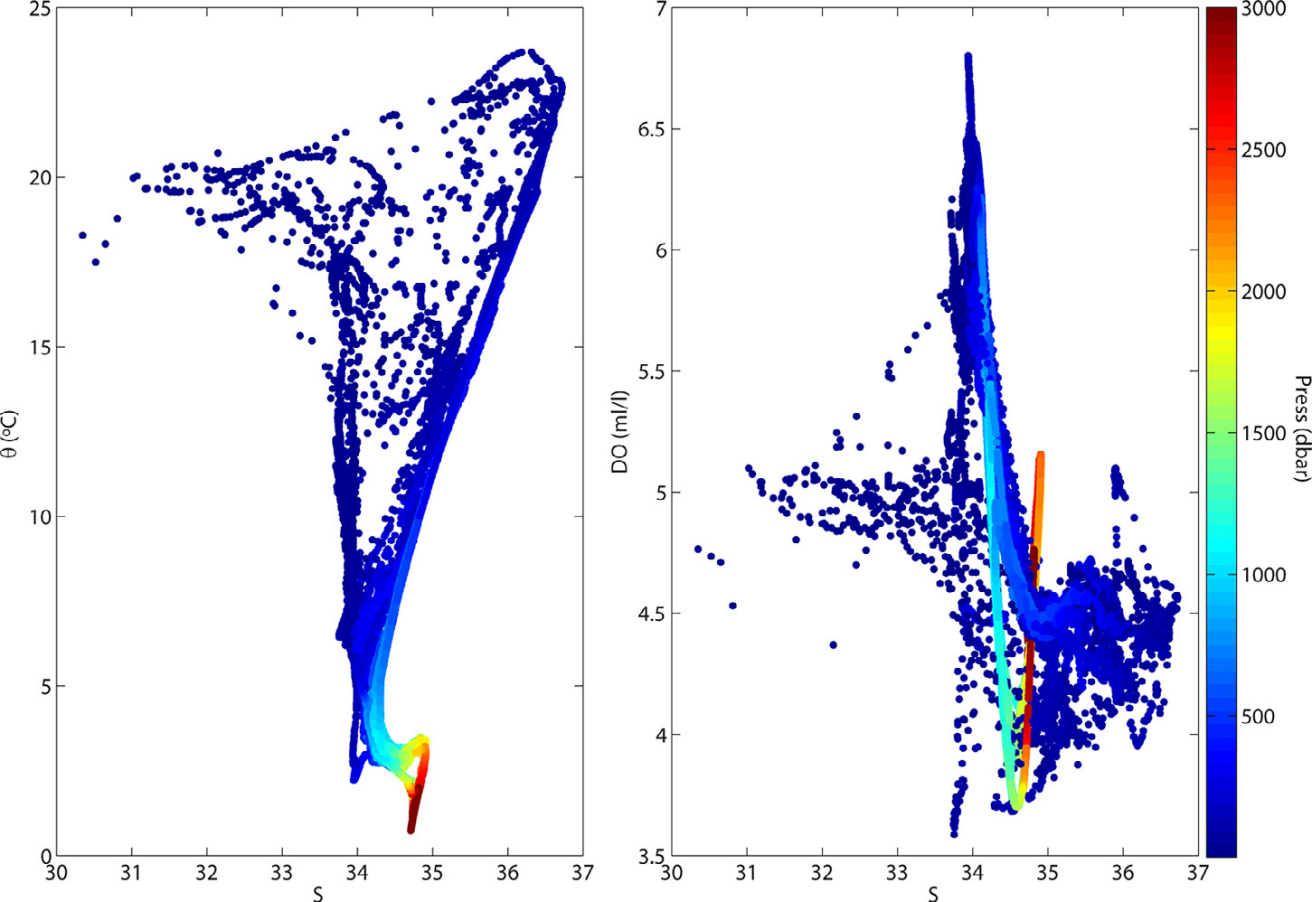
(Left) Potential temperature – salinity (θ-S) and (right) dissolved oxygen – salinity (DO-S) diagrams, color-coded with pressure (dbar).

**Fig. 3:**
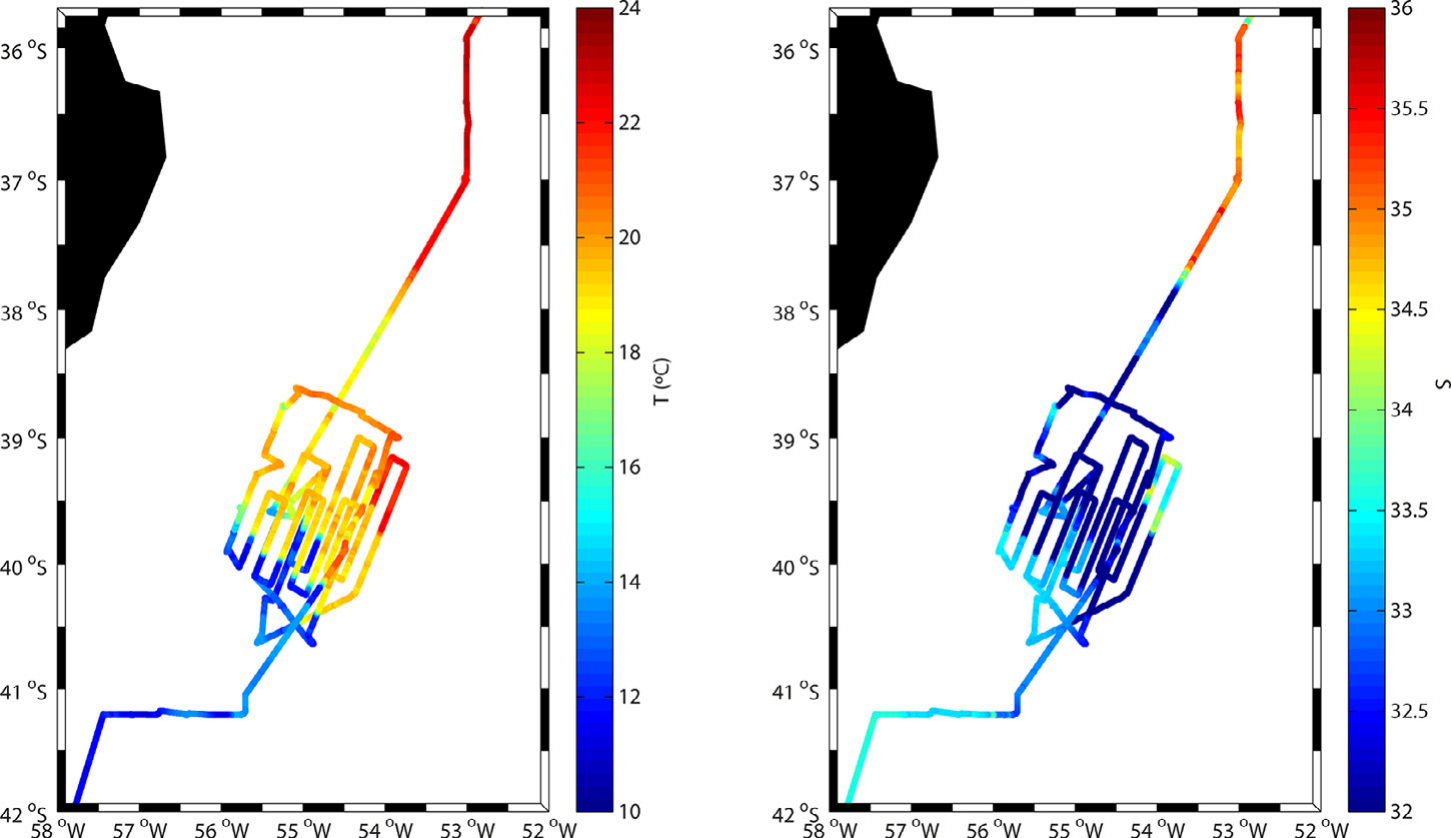
(Left) Temperature (°C) and (right) salinity at 5 m along the cruise track as measured by the vessel's thermosalinograph.

**Fig. 4:**
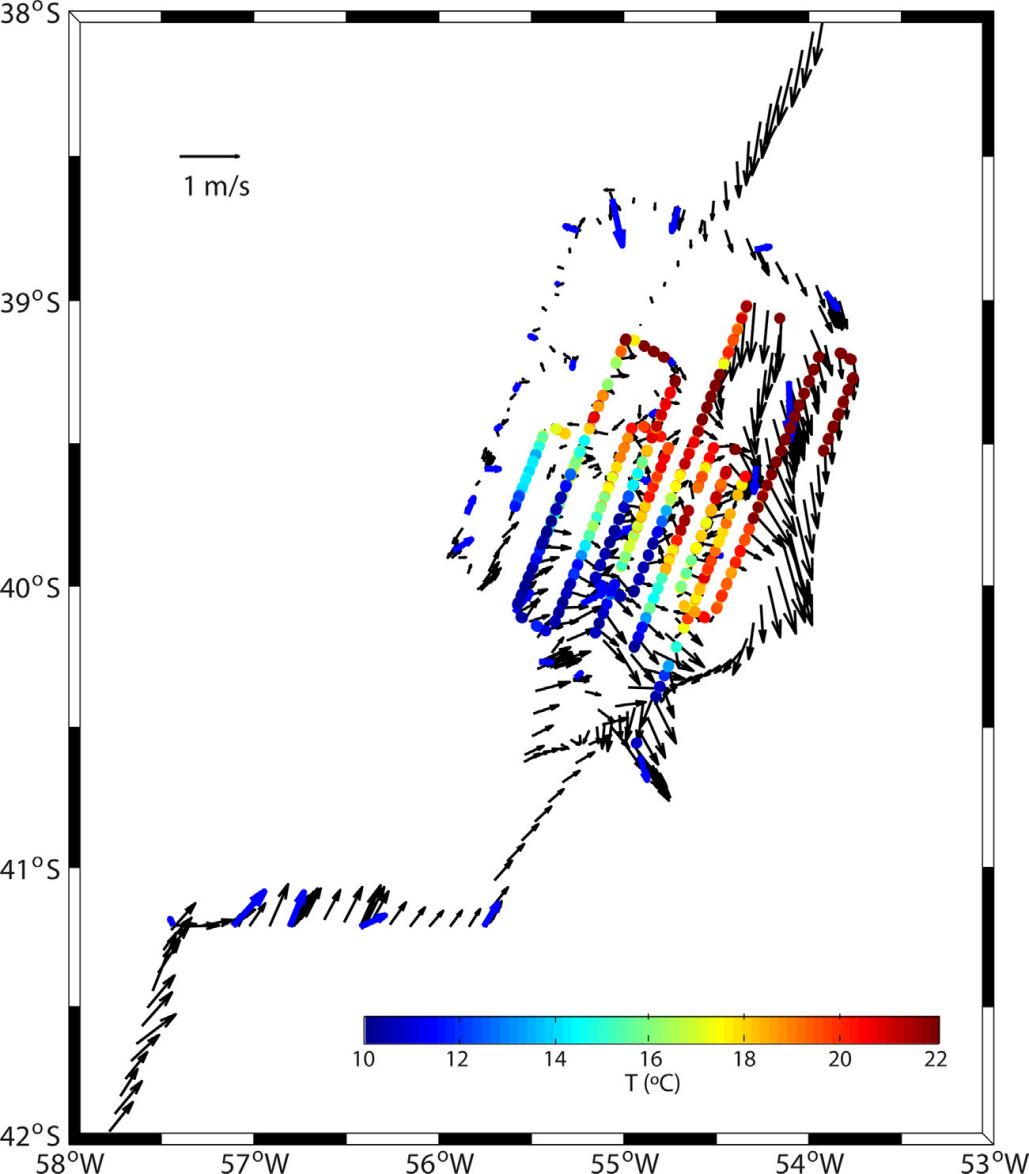
Mean velocity in the upper 800 m of the water column, as obtained with the VADCP (black vectors, plotted every half an hour), and velocity at 4 m for each hydrographic station, as obtained with the LADCP (blue vectors). The average temperature at 22–28 m, as measured during the SeaSoar surveys, is also shown (color-coded). (For interpretation of the references to color in this figure legend, the reader is referred to the web version of this article.)

**Fig. 5:**
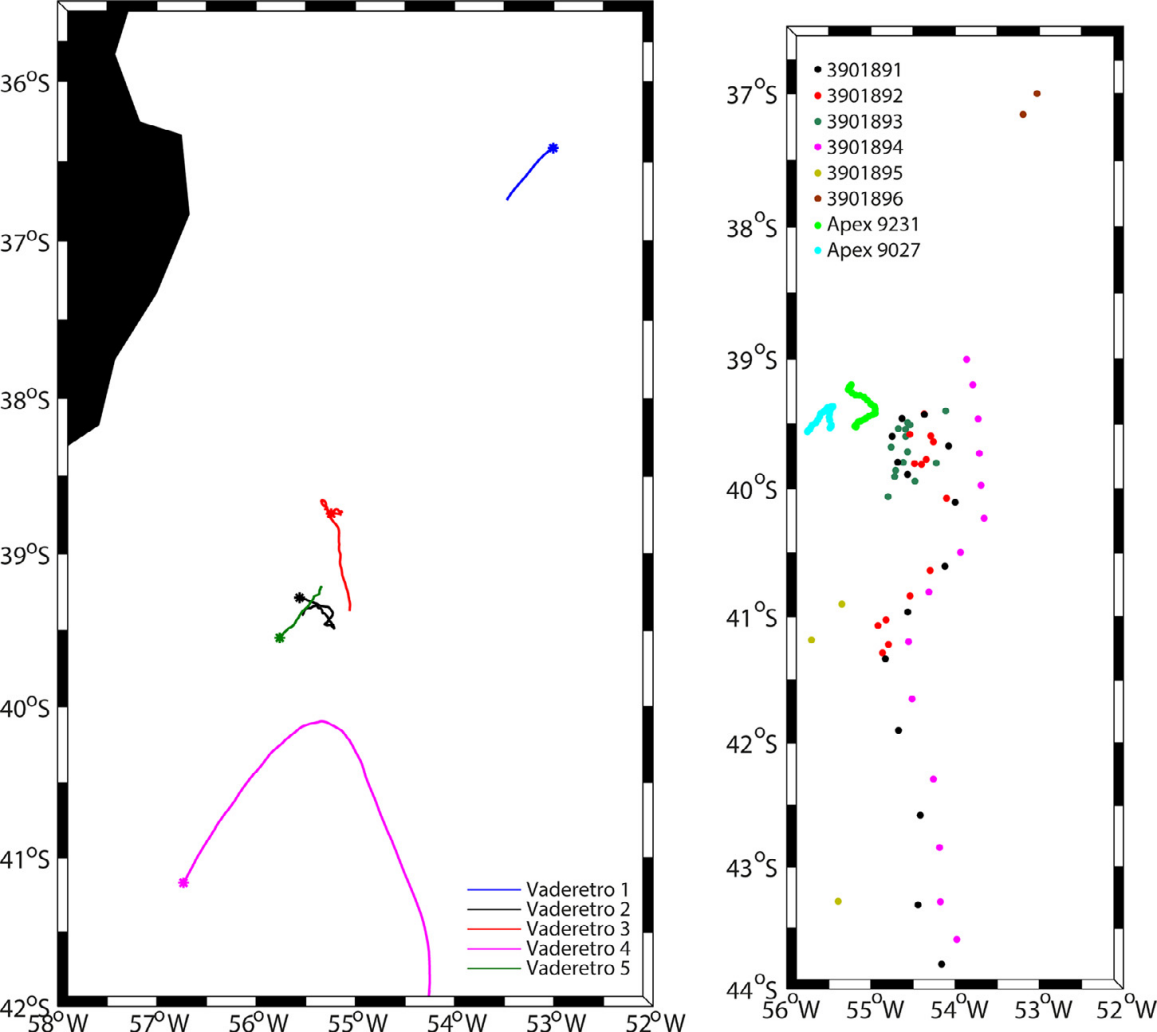
(a) Trajectories of the surface drifters between their launching and 26 April 2017, with the deployment point indicated by a circled star. (b) Location of the profiles carried out by the high-frequency ARVOR-I floats and the APEX 9231 and APEX 9027 floats.

This dataset presents the observations gathered during an oceanographic cruise (RETRO-BMC) in the Brazil-Malvinas Confluence. The data include: (1) continuous along-track near-surface salinity and temperature (Fig. 3), and velocity fields at depths from 20 to 800 m (Fig. 4); (2) 33 hydrographic stations, each consisting of a vertical profile with a multi-parametric probe, a velocity sensor, and a 24-bottle carrousel to collect water samples at discrete vertical positions (Figs. 1b and 2); (3) microstructure measurements in 11 stations (Table 3); (4) two high-resolution surveys carried out with a towed instrumented undulating vehicle (SeaSoar) (Fig. 4); (5) eight high-frequency vertically-profiling instruments, six of them released as part of the international Argo program (http://www.argo.net) and reaching down to either 1800 or 2000 m and the other two released during the time of the cruise and reaching down to typically 500 m (Figs. 1b and 5b; Table 1); and (6) positioning data from five near-surface drifting buoys (dragged at a nominal depth of 100 m) for the time of the cruise (Fig. 5a; Table 2). Here, we present figures and tables that summarize the data available at https://digital.csic.es/handle/10261/188363.

**Table 1:** Deployment and recovery times and positions for the APEXi profiling floats.

Float	Start			End		
	Time and day (yyyy/mm/dd hh:mm:ss)	Latitude S	Longitude W	Time and day (yyyy/mm/dd hh:mm:ss)	Latitude S	Longitude W
9027	2017/04/16 19:16:54	39° 33.60´	55° 44.70´	2017/04/23 12:34:11	39° 31.74´	52° 57.78´
9231	2017/04/16 11:12:19	39° 14.76´	55° 17.58´	2017/04/23 09:00:40	39° 31.08´	55° 11.46´

**Table 2:** Deployment times and positions for the near-surface drifters.

Name	Day (April 2017)	Time (GMT)	Latitude S	Longitude W
Vaderetro1	24	20:37:33	36° 24.85´	53° 00.38´
Vaderetro2	16	12:30:01	39° 17.14´	55° 33.75´
Vaderetro3	16	02:14:11	38° 44.44´	55° 14.58´
Vaderetro4	13	20:17:06	41° 10.06´	56° 43.87´
Vaderetro5	16	17:51:35	39° 32.82´	55° 45.68´

## 2 Experimental design, materials, and methods

The RETRO-BMC survey observations were collected onboard R/V Hespérides in April 2017, departing from Ushuaia (Argentina) on 8 April and ending in Santos (Brazil) on 28 April, on the framework of the VA-DE-RETRO project. The study area was the Brazil-Malvinas Confluence (BMC), at the time of the cruise comprising the region offshore the South American continental platform within 41.2°S–35.9°S and out to 53.0°W (Fig. 1).

Prior to the cruise, the frontal system was positioned thanks to daily sea surface temperature (SST) and sea surface height (SSH) images together with one-week forecasts of temperature, salinity and horizontal velocity down to 1000 m, as provided by the MERCATOR Ocean PSY4V3R1 operational model (1/12° resolution) (http://marine.copernicus.eu). The BMC region was sampled between 13 and 24 April 2017.

The instruments configuration and the cruise planning, including the deployment of the instruments and the launching of the floats and drifters, were done such as to allow an unprecedented multi-scale analysis of this frontal system: from the regional scope and the mesoscale, assessed through continuous along-track sampling and hydrographic stations, to the horizontal submesoscale and the vertical fine-structure, evaluated with the help of an undulating vehicle, and to the microscale structures, studied with a free-falling microstructure profiler.

### 2.1 CTD and continuous measurements

A total of 33 hydrographic stations were done. In 26 of these stations, the water depths were less than 2000 m and the CTD cast reached down to the seafloor. In the other seven stations the sampling reached down to 2000–3500 m.

One core instrument in the hydrographic stations was a SBE 911 Plus multi-parametric probe with a pressure gauge and redundant temperature and conductivity sensors. The probe had attached dissolved oxygen (SBE 43), fluorescence and turbidity sensors (Wetlabs AFL-NTU-RTD). The vertical profile obtained with this probe is commonly known as a CTD cast, standing for the conductivity-temperature-depth measurements.

The probe was mounted on the lower portion of a 12-L 24-Niskin-bottles rosette, which descended and ascended at typical speeds of about 1 m s^−1^. The probe sampled at a rate of 24 measures per second, which was vertically averaged at 1 dbar pressure intervals using the Sea-Bird Electronics Data Processing software. There are both downcast and upcast profiles but, as is standard for CTD casts, we recommend using the downcast simply because of the location of the sensors, which allows sampling the water column before the rosette generates any significant turbulence.

The rosette collected water samples in all stations. The water samples were taken during the upcast, at standard depths plus possibly several other levels of potential interest, identified during the downcast. The water samples were used for several biogeochemical analyses, including the determination of inorganic nutrient concentrations (see below).

All property vertical profiles were first visually checked to detect possible instrument anomalies. During the first two stations there was a clear drift in the primary conductivity sensor (noticeable by comparing the two sensors as well as the downcast-upcast profiles). After the adjustment of the probe connector, the problem was solved in the successive stations. Anomalies were also checked through property-property diagrams, such as the standard potential temperature – salinity (θ-S) diagrams (Fig. 2). The θ-S diagrams confirm that the entire data set is located in a domain that lies between the contrasting subtropical and subantarctic water types (Fig. 2, left). When using other properties, such as dissolved oxygen (DO), the separation between the two water masses may take very different forms (Fig. 2, right).

Additionally, during the entire 1600 nm of the vessel's track in the BMC, a SBE 21 SeaCAT thermosalinograph underway system recorded temperature and salinity at a depth of about 5 m in a continuous mode (one data group every 6 s) (Fig. 3). The data was displayed visually and helped identify when the vessel was crossing the frontal system, characterized by temperature gradients as sharp as 0.2 °C km^−1^.

### 2.2 LADCP and VADCP data

The velocity fields were sampled with two different types of acoustic Doppler current profilers (ADCP). The first one was a lowered-ADCP (LADCP), mounted on the rosette frame, which allowed gathering profiles of horizontal velocity on each cast (Fig. 4). It consisted of a dual-head set-up (down-looking master, up-looking slave) four-beam RDI Workhorse Monitor with a working frequency of 300 kHz, set to obtain velocities in 4-m bins. Two configurations were used: one for casts reaching the sea bottom, which used staggered pings in order to avoid previous-ping interference, and another for profiles not reaching the sea bottom. In those stations down to the seafloor, an altimeter on the lowered package detected the distance between the instrument and the sea bottom, which allowed sampling until about 10 m above the ground. The LADCP data were processed with the Matlab LDEO IX toolbox [1], which uses CTD, vessel's navigation and bottom-tracking data.

The second velocity sensor is the vessel-mounted ADCP (VADCP), an Ocean Surveyor Broadband/Narrowband 75 kHz Teledyne RD Instrument mounted on the hull of the vessel. This equipment allowed gathering velocity data in a continuous mode along the vessel's track, between about 24 and 800 m at 8-m bins (Fig. 4). The instrument was calibrated using its water- and bottom-tracking settings [2] and the raw data were processed with the Common Oceanographic Data Access System (CODAS) [3].

### 2.3 SeaSoar undulating vehicle

Two high-resolution surveys of the frontal system were completed on 17–19 and 19–21 April, respectively, with a Chelsea Group Technologies towed undulating vehicle (SeaSoar) (Figs. 1b and 4). The objective of this repeated high-resolution sampling was to assess the spatiotemporal variability of the BMC front, with special focus on the thermohaline intrusions [4]. With this goal, each survey was designed onboard, continuously changing the location and length of the meridional transects according to in-situ continuous measurements provided by the thermosalinograph, VADCP and the SeaSoar itself. Grossly, each survey consisted of six cross-frontal near-parallel transects, each about 100 km-long. The first survey (SeaSoar1) was completed within 46 h, covering 620 km, while the second one (SeaSoar2) started 40 h after and sampled 751 km during 52 h.

The SeaSoar was equipped with a SBE 9 Plus CTD, with pressure, redundant temperature and conductivity sensors, and additional fluorescence and DO sensors. This equipment recorded data, while pulled by the vessel at a sustained speed of 8.5 knots, optimally undulating in a sawtooth pattern between 5 and 360 m depth, with a horizontal spacing between apogees of 4 km. This range was generally completed except when changing the vessel's heading, where the vertical span was reduced, and the upper and lower meters were lost. The distance between cross-frontal tracks in each of the surveys is 10 nm, with both survey-tracks interlaced in such a way that the spacing between tracks was 5 nm.

### 2.4 High-frequency profiling floats

During the cruise, six NKE Instrumentation ARVOR-I profilers from the Euro-Argo program (https://www.euro-argo.eu/) were deployed, all them with parking depth at 1000 m (Figs. 1b and 5a). Two of them were set up following the Argo standards, i.e. completing a temperature and salinity profile between the surface and 2000 m every 10 days. The other four, launched at the frontal system, had a high-frequency cycling, completing one profile between the surface and 1800 m per day for the first 20 days, thereafter recovering the standard configuration cycle. These are identified by the platform numbers 3901891–3901896 of the World Meteorological Organization.

Moreover, two APEXi profilers (Teledyne Webb Research) were also launched (Figs. 1b and 5b; Table 1). Their cycle-time and parking depth configuration were controlled from the vessel in real time thanks to their Iridium transmitters with bidirectional communication. During the 8 days they remained in the water, these two profilers completed a total of 53 profiles, in most of them between the surface and about 500 m. Each of them was equipped with a SBE 41CP CTD. Further, APEX float 9027 had additional fluorescence and DO sensors.

### 2.5 Drifting buoys

Five subsurface drifting buoys were launched (Figs. 1b and 5a; Table 2). These drifters consist of a spherical surface buoy, containing the batteries and the electronics of the system, and a 15-m long and 1-m diameter holey sock dragged at a nominal depth of 100 m [5]. Each buoy was equipped with a global positioning system and a receiver/transmitter from Global Star satellites. Positions were acquired every hour.

### 2.6 Microstructure profiles

**Fig. 6:**
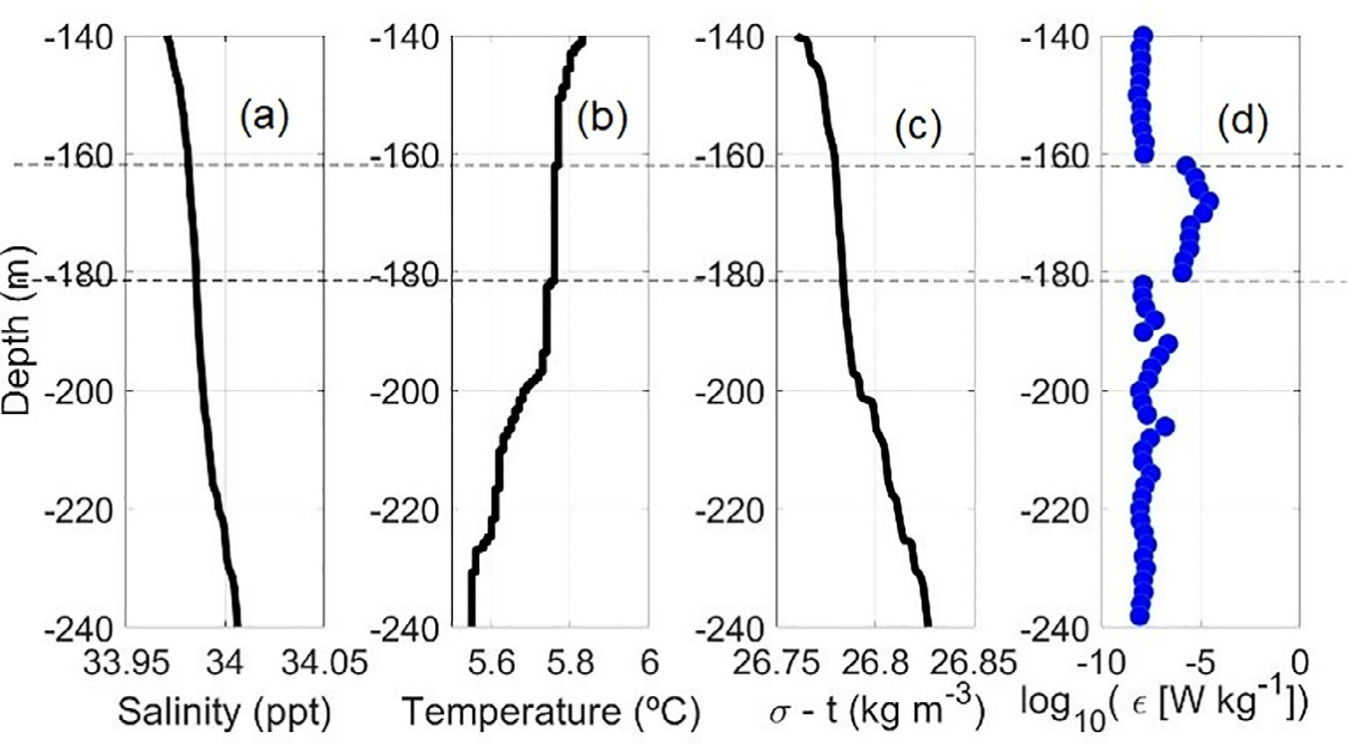
(a–c) Example of the salinity, temperature and density profiles between 140 m and 240 m in a microstructure station, and (d) the corresponding dissipation rate profile as obtained for 2-m segments.

Microstructure profiles were gathered with a free-falling vertical microstructure profiler MSS90L (Sea & Sun Technology) carrying two small-scale shear probes and precision CTD sensors, all them calibrated by the manufacturing company before the cruise. A total of 36 profiles were collected in 11 CTD stations. A minimum of two (except in station CTD010 with only one because of an operational difficulty) and up to seven microstructure profiles were collected at each single station, extending from the surface to between 160 m and 400 m (Fig. 1b; Table 3). The instrument provides in situ temperature (*T*), salinity (*S*) and kinetic dissipation rate (*ε*) as a function of pressure (Fig. 6).

**Table 3:** Microstructure profiles and maximum depth reached at each station.

CTD station	Microstructure profiles	Maximum depth (m)

CTD003	MSS003-MSS007	300
CTD006	MSS008-MSS011 & MSS014	160
CTD009	MSS015-MSS018	280
CTD010	MSS019	195
CTD019	MSS020-MSS023	270
CTD021	MSS025-MSS027	300
CTD022	MSS028-MSS030	360
CTD023	MSS031-MSS032	320
CTD026	MSS035-MSS039	400
CTD032	MSS040-MSS041	380

**Fig. 7:**
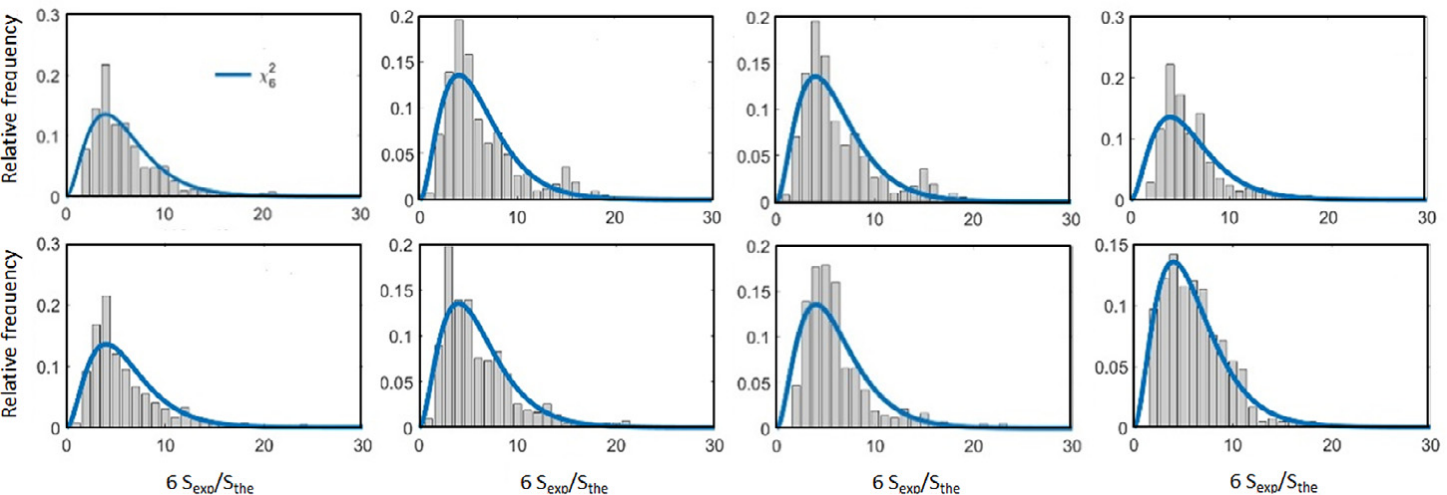
Example of histograms of the ratio of the spectra derived from the recorded small-scale shear data (S_exp_) and computed with the Panchev–Kesich model (S_the_), and the theoretical $\chi_{6}^{2}$ probability distribution functions (blue line), for the eight first 30-m segments in one single profile. (For interpretation of the references to color in this figure legend, the reader is referred to the web version of this article.)

The data quality of the small-scale shear was tested by comparing their spectral representation (experimental spectra) with the theoretical model [6]. A best-fit *ε* was inferred by adjusting the experimental spectra to the one-dimensional transversal Panchev–Kesich shear spectra [7], [8] in the 6–20 cpm range using the maximum likelihood spectral method [9]. By comparing the ratio of the experimental spectra and the Panchev–Kesich theoretical spectra, we verified that the statistical variability of the fit follows a chi-squared distribution with 6 degrees of freedom, $\chi_{6}^{2}$, which was then used to obtain the likelihood function (Fig. 7).

### 2.7 Inorganic nutrients

At each hydrographic station, 50-ml water samples were obtained from the Niskin bottles. These water samples were gathered at standard water depths plus a selected number of depths, which changed depending on the maximum sampling depth and the observation of particular features during the descending CTD cast; two replicate samples were taken at each of these depths.

Samples were immediately frozen at −20 °C and analysed within three months at the Institut de Ciències del Mar in Barcelona using an AA3 HR Seal Analytical instrument, following the methodology and with the same data limits and accuracies as described in [2], [10], [11].
